# Integrating Comorbidities and Phenotype-Based Medicine in Patient-Centered Medicine in COPD

**DOI:** 10.3390/jcm9092745

**Published:** 2020-08-25

**Authors:** José Luis Lopez-Campos, Borja Ruiz-Duque, Laura Carrasco-Hernandez, Candelaria Caballero-Eraso

**Affiliations:** 1 Unidad Médico-Quirúrgica de Enfermedades Respiratorias, Instituto de Biomedicina de Sevilla (IBiS), Hospital Universitario Virgen del Rocío/Universidad de Sevilla, 41013 Sevilla, Spain; borja_994@hotmail.com (B.R.-D.); lauracarrascohdez@gmail.com (L.C.-H.); ccaballero-ibis@us.es (C.C.-E.); 2 Centro de Investigación Biomédica en Red de Enfermedades Respiratorias (CIBERES), Instituto de Salud Carlos III, 28029 Madrid, Spain

**Keywords:** COPD, clinical phenotypes, comorbidities, personalized medicine

## Abstract

Despite recent notable innovations in the management of chronic obstructive pulmonary disease (COPD), no major advances in patient-centered medicine have been achieved. Current guidelines base their proposals on the average results from clinical trials, leading to what could be termed ‘means-based’ medical practice. However, the therapeutic response is variable at the patient level. Additionally, the variability of the clinical presentation interacts with comorbidities to form a complex clinical scenario for clinicians to deal with. Consequently, no consensus has been reached over a practical approach for combining comorbidities and disease presentation markers in the therapeutic algorithm. In this context, from the patients’ first visit, the clinician faces four major dilemmas: (1) establishing the correct diagnosis of COPD as opposed to other airway diseases, such as bronchial asthma; (2) deciding on the initial therapeutic approach based on the clinical characteristics of each case; (3) setting up a study strategy for non-responding patients; (4) pursuing a follow-up strategy with two well-defined periods according to whether close or long-term follow-up is required. Here, we will address these major dilemmas in the search for a patient-centered approach to COPD management and suggest how to combine them all in a single easy-to-use strategy.

## 1. Introduction

Over the last few decades, different approaches have been adopted to achieve a single therapeutic algorithm that is both able to capture the complexity of chronic obstructive pulmonary disease (COPD) and simple enough to apply to general medicine in everyday clinical practice. Notably, the rationale for identifying patient types is based on either the requirement for a specific therapeutic approach or the evaluation of a particularly relevant clinical impact in terms of disease burden or prognostic impact [1]. Unfortunately, at the start of this new decade, this unified approach has not yet been achieved and there are still a considerable amount of controversy to overcome [2]. In this regard, current approaches establishing medical care according to risk level [3] or phenotype [4] have not yielded universally adopted approaches, and have given rise to major controversy [5]. However, with a high prevalence in the population [6,7] and a considerable burden on patients and the health system, COPD is a condition in which more personalized medical treatment should be given.

If we evaluate the literature on the differentiating factors of clinical presentation or therapeutic response in COPD, the variables associated with a specific therapeutic response can be summarized in two groups: variables related to disease presentation, termed as clinical phenotypes [8], and those related with comorbidities [9]. Remarkably, despite the recognized importance of these variables, no practical approach has been agreed on how to combine comorbidities and disease presentation markers into the therapeutic algorithm. One of the main problems with current guidelines is that they base their recommendations on the average results of clinical trials, leading to what we might call ‘means-based’ medical practice. However, the therapeutic response is variable to specific patients, so it is impossible to predict that a given patient will respond like an ‘average’ patient. This is what is termed in epidemiology the ecological fallacy, which implies that we cannot attribute the therapeutic response of a whole cohort to a single patient. In this review, we aim to revise the current knowledge to identify which variables from clinical phenotypes and comorbidities should be considered in a patient-centered approach to COPD management and how to combine them all in a single easy-to-use strategy. Our proposal is to use a two-step approach, using clinical presentation to decide on the initial treatment, and combine them with comorbidities for non-responders, by trying to answer some of the clinical dilemmas.

## 2. The First Dilemma: Does the Patient Have COPD

Despite the fact that the guidelines for recommendations on the diagnosis of chronic airway diseases are clear in their diagnostic criteria, in everyday clinical practice, when the patient goes to their clinician for the first time, there are several issues that must be addressed in the initial differential diagnosis. One of the most important nuances in the diagnosis of chronic airway diseases is that they present a very limited number of clinical symptoms, which in most patients with an airway disease include dyspnea, cough, expectoration, chest tightness, fatigue and limitation in their daily activities. Thus, frequently, the difference between the different diseases is more in terms of distribution, frequency and triggers of these symptoms, than in the presence of these symptoms themselves. This nuance is especially relevant when the clinician has to differentiate between the two most prevalent airway diseases: asthma and COPD [10]. The key markers identifying the diseases in the current international guidelines for asthma [11] and COPD [12] are summarized in Table 1. The difficulty is even greater if we consider that the clinical difference between the two diseases is not as clear as the guidelines state, and a number of patients can present clinical symptoms and functional behavior which overlap between asthma and COPD [13]. This old debate between the two diseases [14] is still going strong today [15] and rose to another level when the terms ‘asthma and COPD overlap syndrome’ or simply ‘asthma and COPD overlap (ACO)’ were coined in the early 2010s [16,17]. Since then, the debate on this overlap has intensified considerably, with 3 major points of controversy.

### 2.1. What Is ACO

On the one hand, intuitively, it could be said that a person with ACO is one who meets the diagnostic criteria of the two diseases and, therefore, has two different diseases. However, on the other hand, the concept of ACO has also been applied to patients with a single disease (asthma or COPD) but who have peculiar clinical behavior which resembles the other disease (Table 1) [5]. This confusion has led to different diagnostic criteria with differences in prevalence and clinical consequences [18,19].

The clinical implications of this double concept are evident, since, if a patient has two diseases, we could potentially apply the treatment for the two conditions (biological therapy, roflumilast, etc.) when they are indicated. However, if there is only one disease with a peculiar or different clinical behavior, then, only the treatments for that single disease could be applied, in the absence of any clinical trials showing us otherwise how to proceed [5]. Until now, this debate has been largely academic, without offering any insight into the clinical care of patients. However, since the advent of biological therapy in asthma or specific phenotype-based therapies in COPD (e.g., roflumilast), the debate has resulted in a genuine clinical problem: if the patient does not have asthma, they will not have access to biological therapy [20,21]. Consequently, clinicians must diagnose with the utmost accuracy in doubtful cases due to the potential therapeutic implications for patient care and the new algorithms to identify ACO which are continually being proposed [22].

To make this decision easier, we can assume a diagnosis of asthma or COPD if both clinical presentation and complementary tests suggest the same diagnosis (Table 1) and identify an ACO as a patient with either a diagnosis of both or a mismatch between the clinical presentation and the results of the complementary tests (Table 1). In many cases, a provisional diagnosis should be established after the first clinical visit, and this should be refined depending on the long-term progression of the disease [23]. In this regard, the GOLD document recommends that the evaluation of the presence or absence of airflow obstruction based on a single measurement of the postbronchodilator forced expiratory volume in 1 s (FEV_1_)/forced vital capacity (FVC) ratio may be misleading and should be confirmed by repeated spirometries, on more than one occasion if the value is between 0.6 and 0.8, as in some cases the ratio may change over time [24,25]. Therefore, consequently, some patients will need follow-up in order to correctly assess clinical and functional behavior and reach an accurate diagnosis.

### 2.2. How to Identify an ACO

Assuming that the clinical presentation of airway diseases is of limited use in identifying ACO, most of the approaches to diagnosing an ACO have been based on the results of different complementary tests, mainly blood eosinophils or broncho-reversibility testing.

Different studies have highlighted the potential role of blood eosinophils in patient selection for specific treatments [26,27,28,29] with a biological background [30]. However, the blood eosinophil count is nonspecific, since it has a weak correlation with eosinophilic inflammation and there are other causes of eosinophilia in COPD patients [31,32,33]. Different approaches have been proposed to use the blood eosinophil count more wisely for treatment selection by including the clinical context [34], persistence over time [35] or the combination with exhaled biomarkers [36,37], which should increase specificity and make it more acceptable in the future.

Some authors suggest using the bronchodilator test to identify patients with ACO [38]. This idea starts from the diagnosis of asthma confirmed by a positive bronchial reversibility in a suggestive clinical context (Table 1). However, the role of the bronchodilator test in COPD is not very clear [39], since bronchodilator reversibility is at least as common in participants with COPD as those with asthma [40]. It is important to keep in mind that a positive bronchodilator test does not necessarily imply bronchial hyperresponsiveness. Not only are bronchodilation and hyperresponsiveness different concepts, but they also lack correlation at the patient level, as has been shown in numerous previous works [41,42].

**Figure 1:**
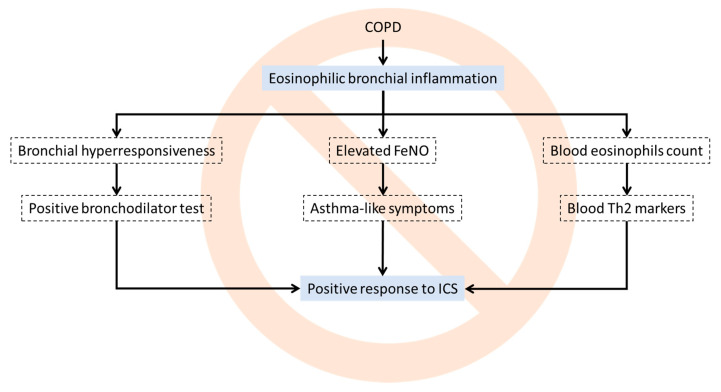
Some erroneous associations that clinicians frequently make when evaluating COPD patients.

Similarly, other markers proposed to identify ACO, such as exhaled nitric oxide [43] or total IgE [44], have not been fully developed clinically to identify this type of patient. The current situation is, therefore, that it is extremely difficult to identify a patient with ACO, and, as a result, clinicians often make mistakes when evaluating ACO in daily clinical practice. This error is derived from the fact that the clinician assumes that having eosinophilic bronchial inflammation is equivalent to having a high blood eosinophil count, which in turn is related to bronchial hyperresponsiveness and a positive bronchodilator test, which is accepted as an indicator of response to inhaled corticosteroids. However, this deductive process is flawed (Figure 1), since we know that, at the patient level, there is no consensus that these variables concur [41,42]. Therefore, we currently have no single biomarker able to identify ACO patients. Future trial will have to elucidate whether a combination of these could serve as a guide to detect inhaled corticosteroids (ICS)-responders in COPD.

### 2.3. How to Treat ACO

Following on from the previous arguments, if we are not sure what an ACO is and we have no biomarker to identify it, we are obviously unable to design clinical trials to guide us on how to treat these patients. What we do know is that pharmacological therapy for COPD is based on long-acting bronchodilators (LABD) [12], that asthma therapy is based on inhaled corticosteroids (ICS), and that asthma should not be treated with LABD alone [11], so the current recommendation, based on little more than common sense, is to treat these cases with ICS. However, the correct dose regimen of the escalation/de-escalation schemes are still to be defined.

## 3. The Second Dilemma: How Do I Start Therapy

To begin with, it is important to bear in mind that non-pharmacological treatments have a profound impact on the symptomatic improvement and prognosis of COPD, often more importantly than many pharmacological approaches. In a global approach to the disease, the two therapeutic options with a clear impact on the prognosis of the disease are smoking cessation and exercise [45,46]. Therefore, it is crucial to convey to the patient that COPD is treated by quitting smoking and exercising as first steps. In this sense, the health system must have available to patients and as part of their comprehensive care measures for smoking cessation and rehabilitation programs of varying intensity and nature according to the characteristics of the disease and available resources [47,48]. Additionally, a balanced diet and a correct vaccination program are also relevant aspects of non-pharmacological treatment [49,50].

**Figure 2:**
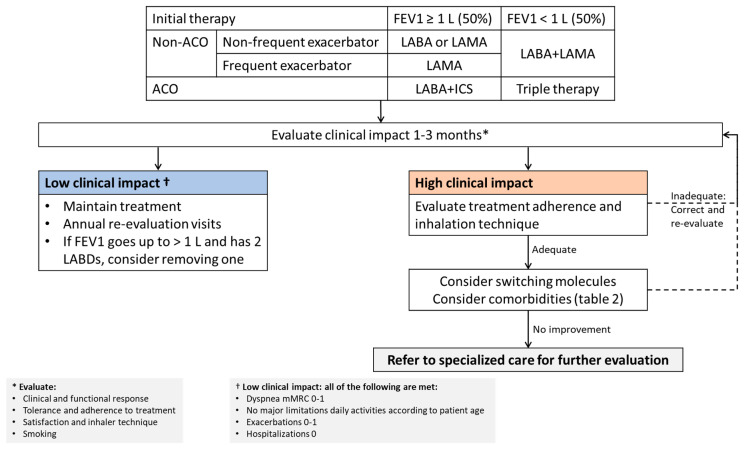
Proposal of initial pharmacological approach.

From a pharmacological perspective, in light of the above arguments, and despite the difficulties mentioned, when establishing the initial treatment for the patient with confirmed COPD, it seems logical to observe whether the patient presents with classic COPD or some form of ACO that requires treatment with ICS. This decision will obviously influence the first treatment. The next step will be to decide whether to start treatment with one or two LABD, basing our decision mainly on the symptoms (mainly dyspnea), the number of exacerbations and lung function measured by spirometry [12]. It is important to bear in mind that these three variables show a very poor correlation with each other at the patient level [51], and will have therefore to be considered separately, since each one provides us with relevant clinical information. Interestingly, despite the unquestionable importance of dyspnea in COPD [52], in the present approach, we propose this variable will have a more prominent role during the follow-up than in the initial therapeutic decision, since untreated symptomatic COPD usually features dyspnea, which improves with bronchodilator treatment. Additionally, numerous studies have demonstrated the benefits of double bronchodilator therapy in COPD, but, curiously, it does not produce a consistent improvement in dyspnea [53], so that very few studies have noted this improvement when compared to a single LABD therapy [54,55]. Similarly, the impact of two LABD vs. one LABD in terms of exacerbation prevention has not been consistently proven in most trials and remains a matter of controversy [56]. However, the decision of the first LABD may be influenced by the existence of a frequent exacerbator, since it has been consistently shown that long-acting muscarinic antagonists (LAMA) are much better at preventing exacerbations than long-acting beta agonists (LABA) [57,58]. Finally, lung function is probably the parameter that best responds to double bronchodilation. This improvement in trough FEV_1_ has been demonstrated in all clinical trials comparing double bronchodilation vs. single bronchodilation [53]. Therefore, since FEV_1_ has clear prognostic implications, it follows that this parameter could help us decide how to start therapy. Despite the recommendations of the GOLD document, lung function should probably be included in the risk stratification of COPD [59]. Probably the most widely accepted cutoff value to identify severe lung function impairment is 1 L, in absolute terms, or 50% [12]. Altogether, with these premises in mind, Figure 2 shows a proposal for an initial pharmacological therapy.

## 4. The Third Dilemma: What If the Patient Does not Respond to Initial Therapy

If the patient does not respond satisfactorily to this therapeutic approach, there are two initial steps that should be carried out before beginning a therapeutic escalation (Figure 2). The first and most important is to verify the most suitable inhalation technique to correct critical errors [60]. Numerous studies have shown that a considerable number of subjects use their devices incorrectly [61]. Therefore, devices need to be matched with the patient and proper education to use these devices correctly is essential [60,62]. Even if it has been done previously, verifying the inhalation technique should be part of the patient’s health education and evaluated in each contact with the health system [62]. Second, we need to verify treatment adherence, which, in the case of inhaled medication, can be problematic. Although problems with compliance may be revealed in a conversation with the patient, more objective questionnaires and systems have been designed to evaluate this variable [63,64].

With these two measures, a significant percentage of patients will improve their results for inhaled therapy. However, if the impact of the disease on the patient remains high, there are two further options (Figure 2). One is to consider changing the treatment for another in the same pharmacological group, as changing the molecule leads to two potential benefits: first, we know that the response to inhaled treatment of certain drugs varies between patients and some patients respond better to certain molecules than to others [65]; second, changing the molecule is often accompanied by a change in the inhalation device, which the patient may handle better, if trained accordingly. Finally, in spite of all these actions, if the severe impact of the disease on the patient persists, they will need to be referred to respiratory specialized care for a more comprehensive evaluation of the disease and its comorbidities, which we will discuss further below.

Patients with persistent symptoms or exacerbations despite following the correct therapy (i.e., pharmacological or non-pharmacological therapies with good adherence and a correct inhalation technique) represent a clinical challenge that should be evaluated by respiratory specialized care, since there are many different conditions that may be influencing the lack of response [66]. As already suggested years ago [67], the evaluation of the patient will depend on what the main problem is, dyspnea or exacerbations, but a considerable number of variables and comorbidities may influence the impact of the disease on any given patient [68,69]. In particular, certain comorbidities must be mentioned due to their possible complexity and interaction [70]. It would make no sense to produce a diagram showing how to escalate inhaled therapies during the follow-up, as previously suggested [12], since the complexity of the interaction between comorbidities precludes that approach. Instead, rather than escalating the therapy blindly, the clinician should evaluate a number of respiratory and non-respiratory conditions that may influence the perception of symptoms or the frequency of exacerbations (Table 2).

Among the respiratory conditions, the impact of smoking on the presentation of the disease and a lack of therapeutic response is of the utmost importance [71]. Predominant emphysema COPD where hyper-inflation plays a major role is worth evaluating for the potential of interventions such as lung reduction surgery or bronchial valves to be considered. Additionally, the association of COPD with asthma is also widely recognized. Interestingly, it has been shown that some patients with other respiratory comorbidities such as bronchiectasis can be associated with bronchial asthma [72]. Chronic bronchial infection should also be ruled out in these cases, particularly in those with persistently frequent exacerbations or purulent sputum.

The presence of potentially pathogenic microorganisms in the airway of COPD patients has several consequences, including a greater impact of symptoms, worse functional progression, and increased risk of exacerbations and worse prognosis [73,74,75]. Lung cancer should also been screened in these patients, not only since it shares common factors with COPD but also because of the well-known association between the two conditions [76]. Currently, two major debates about the role of active lung cancer screening by CT scan [77,78] and the potential role of ICS on lung cancer prevention [79,80,81] are driving the research in this context. Sleep apnea can also mimic a high disease burden in COPD patients, and this should be correctly screened [82]. Another condition influencing the disease burden is the combination of emphysema and lung fibrosis, which may have a complex interaction in lung mechanics [83,84]. To sum up, it follows that a dedicated clinical interview specifically exploring asthma and sleep apnea, together with imaging techniques and sputum culture should be part of the systematic approach in persistently symptomatic or exacerbated patients.

Although a thorough revision of all the comorbidities lies beyond the scope of this review, a number of comorbidities should be considered when evaluating a non-responding COPD patient (Table 2). Among the systemic conditions, cardiovascular disease leads the ranking [85]. The physiologic interaction between the two organs [86], the overlap between some clinical expressions of cardiovascular diseases with COPD [87], the shared risk factors [88] and the influence of treatments on each other [89,90], all underline the need to explore this association in persistently symptomatic COPD patients. Interestingly, many cases of cardiovascular conditions remain undiagnosed in COPD [91]. Osteo-skeletal conditions also produce a major impact on the patients, with osteoporosis and vertebral fractures exerting a profound impact on COPD [92]. Additionally, skeletal muscle dysfunction represents another comorbidity worth considering that should be explored with suitable tools [93,94], and physical activity should be actively encouraged in these cases [95,96]. Other conditions including malnutrition [97], mood disorders [98], hematologic disorders [99], gastro-esophageal reflux [100] or vitamin D deficiency [101], among others, have been consistently associated with a high burden of COPD either in the form of persistent symptoms or exacerbations [66,102].

Unfortunately, although all these comorbidities are closely linked to COPD and may influence disease presentation, no algorithm to study them all in a systematic, organized, cost-effective way has been officially proposed. Similarly, to date, no initiatives have been put forward to assess the best algorithm to study respiratory and non-respiratory comorbidities in COPD patients with a high impact of the disease. We recommend starting with a thorough systematic medical interview to explore the presence of these comorbidities, including (at least) an ECG/echocardiogram, nutritional evaluation, thyroid function, vitamin D, blood cell count and a 24 h esophageal pH test as complementary tests, followed by individualized studies. Those cases that persist with a high clinical impact (Figure 2) despite taking all the measures available at each level of care (Table 2), should be referred for evaluation of specialized pulmonary care.

## 5. The Fourth Dilemma: How Do I Organize Follow-Up

**Figure 3:**
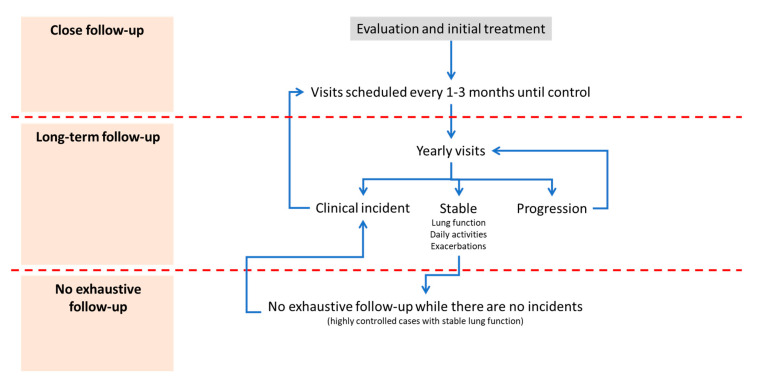
Different stages of monitoring the patient with COPD.

Finally, such a diversity of therapeutic options must be linked to a follow-up scheme that is both conscientious and flexible, allowing patients to be given simple and adaptable therapeutic schemes for each clinical situation. As a result, it makes no sense to devise a single monitoring scheme for all patients, but a general scheme should be set up in which all patients can be included. A general idea for this is put forward in Figure 3, which identifies two types of follow-up after the initial treatment. On the one hand, after selection of the initial treatment, a rigorous follow-up is initially required, every few months, to evaluate the initial therapeutic response, assess possible adverse effects, correct problems with the inhalation technique and evaluate patient satisfaction with the treatment.

Once these objectives have been achieved, the monitoring should change its objective completely, to that of evaluating the long-term progression of the disease. The goal here is not so much symptomatic relief (which has presumably been achieved in the previous phase) as intervention in the progression of the disease. For this reason, visits at least annually will be needed to assess whether the progression has stopped, especially in terms of lung function or impact of the disease. Naturally, when exacerbations or incidents occur that require a rapid change in treatment, we must return to the program for a closer, temporary follow-up. If, after a few years of follow-up, the disease does not worsen functionally and maintains a low impact, we may consider discontinuing the active follow-up of these patients provided that no new clinical incidents occur.

## 6. Conclusions

The treatment of patients with COPD in a more personalized way must address diverse aspects not only related with the disease, but also with its comorbidities, and current schemes do not offer such personalized medical treatment. However, it is possible to design an approach based on the specific patient rather than on ‘means-based’ medical practice. To overcome this limitation, it is important to think outside the box and use the available evidence to propose new algorithms that help the clinician in decision-making. In this context, we understand that each patient should be evaluated in light of both the clinical presentation and their relationship with comorbidities: only then will it be possible to achieve a more patient-centered assessment.

## Figures and Tables

**Table 1:** Markers of asthma and chronic obstructive pulmonary disease (COPD) according to GINA (Global Initiative for Asthma) [11] and GOLD (Global Initiative for Obstructive Lung Disease) documents [12].

Asthma	COPD
**Clinical presentation (for suspicion)**	
**Symptoms:** Wheezing Shortness of breath Chest tightness Cough	**Symptoms:** Dyspnea Cough Sputum production Wheezing Chest tightness Weight loss
**Distribution of symptoms:** Generally more than one Occur variably over time Vary in intensity Often worse at night or on waking Often triggered by exercise, laughter, allergens, cold air Often appear or worsen with viral infections	**Distribution of symptoms:** Chronic and progressive May vary from day-to-day May precede the development of airflow limitation by many years
**Complementary tests (for confirmation)**	
Documented excessive variability in lung function with documented expiratory airflow limitation, with any of the following: Positive bronchodilator (BD) reversibility test Excessive variability in twice-daily PEF over 2 weeks Significant increase in lung function after 4 weeks of anti-inflammatory treatment Positive exercise challenge test Positive bronchial challenge test Excessive variation in lung function between visits	Spirometry is required to make the diagnosis in this clinical context; the presence of a post-bronchodilator FEV1/FVC < 0.70 confirms the presence of persistent airflow limitation and thus of COPD in patients with appropriate symptoms and significant exposures to noxious stimuli.

**Table 2:** List of studies that should be carried out in patients with persistent symptomatic COPD.

Comorbidities	Complementary Test
**Respiratory conditions**	
Bronchial asthma	Re-evaluation of asthma diagnostic criteria
Bronchiectasis	High-resolution CT scan
Chronic bronchial infection	Sputum culture
Lung hyperinflation	Lung volumes and diffusing capacity High-resolution CT scan
Alpha1-antitripsyn deficiency	Serum alpha1-antitrypsin, if not yet done
Lung cancer	Chest radiography/CT scan
Pleural effusion	Chest radiography
Sleep apnea	Sleep study
Interstitial lung disease (emphysema-fibrosis)	High-resolution CT scan
**Non-respiratory conditions**	
Cardiovascular disease	Electrocardiogram + echocardiography Cardiopulmonary exercise testing
Muscle deconditioning	Exercise testing
Malnutrition	Evaluation of nutritional status
Mood disorders	Mood disorder screening
Anemia/polyglobulia	Blood cell count
Hypothyroidism	Thyroid function
Liver insufficiency	Liver function
Kidney insufficiency	Kidney function
Gastro-esophageal reflux	Endoscopy 24 h esophageal pH test
Vitamin D deficiency	Vitamin D measurement
Primary or secondary immune deficiencies	Immune function analyses
